# Celiac disease: can we avert the impending epidemic in India?

**Published:** 2011-01

**Authors:** B.S. Ramakrishna

**Affiliations:** Department of Gastrointestinal Sciences, Christian Medical College, Vellore 632 004, India rama@cmcvellore.ac.in

Celiac disease, caused by allergy to gluten present in wheat and related grains, is a disorder of considerable and increasing importance in Western countries^1^. Originally recognized primarily in children presenting with diarrhoea and malabsorption, we now understand that it often affects adults, that it may present primarily with non-gastrointestinal manifestations including anaemia, arthropathy, osteoporosis and growth retardation, and what we see clinically is the tip of an iceberg that threatens to grow bigger. The diagnosis of celiac disease is now made with serological tests which may capture those at risk as well as those with actual disease. While small bowel mucosal biopsy is considered essential to the diagnosis of disease, there is now increasing recognition that even a positive serological test is associated with increased risks for vascular disease.

The estimated population prevalence of diagnosed celiac disease in many Western countries approaches 1 per cent^2^–^5^. The highest prevalence rates of the disease are noted in the Saharawi people of Africa in whom celiac disease prevalence is believed to be around 5 per cent^6^. There is little information on celiac disease prevalence in South Asia, and indeed celiac disease is believed to be rare in this part of the world^7^. There is a strong genetic tendency, 75 per cent of monozygotic twins are concordant for the disease, compared to 10 per cent of dizygotic twins; 10 per cent of first degree family relatives of an index patient have celiac disease^8^. HLA alleles encoding the antigens DQ2 and DQ8 show the strongest association with celiac disease; however, most individuals expressing DQ2 or DQ8 do not develop the disease^8^. Multiple other genes contribute to the disease each having a weak effect. Recent genome wide studies show that, in addition to HLA locus genes, the second strongest association is for an SNP close to the IL2 and IL21 genes on chromosome 4q27. Six of 7 additional loci are close to the immune response genes^9^. Geographic differences in disease prevalence are explained to an extent by genetic differences between populations. For instance, carriage of DQ2 and DQ8 is up to 40 per cent in Caucasians and there is a very high carriage of DQ8 among the Saharawi. On the other hand, DQ2 is absent in the population of Burkina-Faso where celiac disease is also absent^10^.

Willem-Karel Dicke recognized the association between consumption of wheat and manifestation of clinical symptoms^11^; during the Second World War when food was in very short supply he observed that children with celiac disease flourished on a diet that did not contain wheat, providing a clue to the aetiology of the disease. It has been realized that the prevalence of celiac disease in a population broadly parallels the amount of wheat consumed in the diet. Thus, celiac disease is low or absent in Japan and southeast Asia where rice is the main cereal consumed and in sub-Saharan Africa where maize is the staple cereal in the diet. Even within Europe, the prevalence of celiac disease is lower in Denmark, Estonia and Finland where lower amounts of gluten are consumed in infancy than in Sweden where gluten consumption during infancy is higher.

The classical presentation of celiac disease, with symptoms referable to the gastrointestinal tract, may account for only a proportion of the cases. We now realize that celiac disease is actually a multi-system disorder which is highly variable in its clinical expression, may occur at any age, and may present with a variety of manifestations^5^. The diagnosis is often delayed for these reasons. Gastrointestinal manifestations may include diarrhoea, weight loss, failure to thrive, stunting, abdominal pain, bloating and distension, anorexia, vomiting and constipation. On the other hand, celiac disease patients often present with extraintestinal manifestations with minimal gastrointestinal symptoms. They may present to the internist or relevant sub-specialist with one or more manifestations including iron deficiency anaemia, stunting, osteoporosis, vitamin deficiencies, or fatigue. They may present to the obstetrician/gynaecologist with delayed puberty, infertility, or recurrent foetal loss. They may present to the dental surgeon with recurrent aphthous ulceration or dental enamel hypoplasia. Celiac disease may be associated with autoimmune endocrinologic disorders such as thyroiditis and type 1 diabetes mellitus. It is associated with neuropsychiatric conditions such as depression, anxiety, peripheral neuropathy, ataxia and epilepsy. Asymptomatic elevation of transaminases may occur in these patients and an unusual association of celiac disease is with chronic liver disease and non-cirrhotic portal fibrosis^12^,^13^.

The diagnosis of celiac disease is based on the presence of serologic tests (ELISA) confirming the presence of antibody to tissue transglutaminase (anti-tTG)^5^. The IgA anti-tTG provides the best specificity and is widely used. However, about 6 per cent of healthy individuals are partially IgA deficient^14^and in these individuals the diagnosis may be missed if only the IgA antibody test is done. Currently available diagnostic kits often provide both IgA and IgG antibody levels, enhancing the ability to make the diagnosis. The presence of positive serology is not in itself sufficient to make a diagnosis of celiac disease. Deep duodenal biopsies, obtained at upper gastrointestinal endoscopy, are often used to confirm the diagnosis by demonstrating infiltration of the epithelium by lymphocytes and the presence of villous atrophy. It also requires the demonstration that gluten withdrawal from the diet will reverse or ameliorate the clinical symptoms. Based on the clinical presentation, serology, and biopsy findings, celiac disease has been classified as classical celiac disease (dominated by gastrointestinal malabsorption), atypical celiac disease (with prominent extraintestinal symptoms and a few or no gastrointestinal symptoms), silent celiac disease (asymptomatic individuals with positive serology and villous atrophy on biopsy) and latent celiac disease (asymptomatic individuals with positive serology but normal biopsy)^5^. Clinical celiac disease is associated with a doubling of all-cause mortality^5^. However, there is now intriguing evidence from serological studies of archived blood that latent celiac disease may also be associated with excess mortality when compared to the normal population. A recent study examined archived blood specimens from a cohort of US Air Force personnel followed for over 45 years and two recent cohorts. Prevalence of anti-tTG antibody and anti-endomysial antibody (*i.e*. undiagnosed or latent CD) increased 4.5- fold from 0.2 per cent in the Air Force cohort to 0.9 per cent in the recent cohorts. At the same time, mortality in individuals with undiagnosed CD was 4 times greater than in those who were seronegative^15^.

Celiac disease was recognized in northern India, primarily in children, since the 1960s^16^–^19^. A community-based study in Ludhiana that involved a step-wise approach to case detection and diagnosis estimated that celiac disease prevalence in this city was at least 1 in 310 individuals^20^. Hospital-based studies examining a general paediatric patient population suggest a prevalence of 1 per cent^21^. Celiac disease affecting adults is also now well recognized in northern India^22^–^25^, and in many of these individuals the presentation is atypical, *i.e*. without diarrhoea or overt malabsorption. The prevalence of celiac disease in southern India is not known. Anecdotal experience of physicians and gastroenterologists in southern India suggests that it is very infrequent in southern India^7^,^26^,^27^.

Differences in celiac disease prevalence between north and south India could be ascribed to differences in dietary patterns (rice being the staple cereal in south India) or due to differences in genetic make-up. Celiac disease occurrence is determined by the HLA- DQ antigen expression pattern of the individual. In particular, almost all patients with celiac disease express either HLA-DQ2 or HLA-DQ8,^1^and these HLA types are also present in about 30 per cent of the normal population. The absence of expression of either HLA-DQ2 or DQ8 in a population is also correlated with the absence of celiac disease in that population. The genes that determine expression of HLA-DQ2 or DQ8 have been studied in many populations. f0 ourteen of the 15 children with celiac disease in Lucknow were HLA-DQ2 positive by serotyping and 29 of 30 children in the same institution were positive in another study^28^,^29^. In another study, 34 of 35 north Indian children with celiac disease were HLA-DQ2 positive^30^. HLA-DQ2 is carried on DR3 haplotypes due to linkage disequilibrium, and the DR3 haplotypes in these 34 north Indian children were different from the DR3 haplotypes described in Caucasian children with celiac disease^30^. The HLA-DQ phenotype of the general population in north and south India is not adequately known. Studies suggest that the prevalence of HLA-DQ2 in a north Indian population is around 32 per cent^31^. There are significant differences in prevalence of HLA-DQ2 in different Indian communities but these studies have all been on limited numbers of individuals, thus not providing confidence in the estimated prevalence^32^–^36^. The other variable is that cereal consumption patterns are very different between north and south India, although there has been a recent change in these patterns particularly in urban areas. In south India, rice is the primary cereal consumed in the diet. In the Indian sub-continent, wheat consumption is high in Pakistan and in the States of north India, which also constitute the celiac belt of India.

The time of first exposure to wheat influences the development of celiac disease. In countries such as Finland, Estonia, and Denmark, characterized by low gluten consumption in infancy, celiac disease prevalence is much lower than in Sweden where gluten consumption is high in infancy. A natural experiment occurred in Sweden about two decades ago when national recommendations were made to introduce wheat into the diet after cessation of breast feeding at six months^37^. This change was coupled with increased wheat gluten consumption through infant feeds. Together these measures resulted in a two-fold increase in incidence of celiac disease in Sweden, which was attributed to introduction of wheat into the diet after cessation of breast feeding. In 1996 this recommendation was changed to introduce gluten in gradually increasing amounts while the infant was still being breast fed. This led to a dramatic decrease in celiac disease incidence. The other dimension to this problem is that not all wheat is alike when it comes to inducing celiac disease. The ancient or diploid wheats (*e.g. Triticum monococcum*) are poorly antigenic, while the modern hexaploid wheats (*e.g. Triticum aestivum*) have highly antigenic glutens, more capable of inducing celiac disease^38^. India, for centuries, grew diploid and later tetraploid wheat which is less antigenic, while hexaploid wheat used in making bread is recently introduced. Thus a change back to older varieties of wheat may have public health consequences. Public health authorities may well want to examine both these avenues, *i.e*. infant feeding recommendations and wheat varieties cultivated in the country, for opportunities to avert the epidemic of celiac disease which is impending in our country.
